# Missed Follow-Up and Kaposi Sarcoma Progression: The Consequences of Medication Non-adherence

**DOI:** 10.7759/cureus.84196

**Published:** 2025-05-15

**Authors:** Kevin Chen, Byron Lee, Stanley Kim

**Affiliations:** 1 Medical School, College of Osteopathic Medicine of the Pacific, Western University of Health Sciences, Pomona, USA; 2 Medical School, College of Osteopathic Medicine of the Pacific-Northwest, Western University of Health Sciences, Lebanon, USA; 3 Division of Hematology and Oncology, Department of Medicine, Kern Medical, Bakersfield, USA

**Keywords:** bictegravir/emtricitabine/tenofovir alafenamide, biktarvy, cabotegravir/rilpivirine, kaposi sarcoma, nonadeherance to medication

## Abstract

Kaposi sarcoma (KS) is a serious AIDS-defining malignancy that can progress rapidly in the setting of profound immunosuppression. Non-adherence to antiretroviral therapy (ART) remains a major barrier to viral suppression, particularly among underserved populations.

We present the case of a 36-year-old African American man who has sex with men (MSM), living in an underserved community, with a history of HIV/AIDS and late latent syphilis. The patient developed disseminated KS after six months of non-adherence to bictegravir/emtricitabine/tenofovir alafenamide (Biktarvy). He presented with a CD4 count of 44 cells/µL and a viral load of 592,000 copies/mL. Clinical findings included multiple violaceous skin lesions, pulmonary reticulonodular infiltrates with pleural effusion, and lymphadenopathy. Biopsy confirmed KS. He was restarted on ART and prophylactic antibiotics upon discharge.

This case highlights the severe consequences of ART non-adherence in a vulnerable population. Targeted interventions, including long-acting ART, behavioral support, and telehealth innovations, are essential to improve retention in care and prevent advanced HIV-related complications like KS.

## Introduction

Kaposi sarcoma (KS) is an angioproliferative malignancy characterized by multifocal tumors that typically affect the skin and mucous membranes, and less frequently, visceral organs. Four epidemiologic variants of KS have been described: classic (sporadic), endemic (African), epidemic (AIDS-associated), and iatrogenic (transplant- or immunosuppression-associated) KS [1,2]. The causative agent of all forms is KS-associated herpesvirus (KSHV), also known as human herpesvirus-8 (HHV-8). KSHV encodes viral proteins that promote immune evasion and cellular transformation, facilitating the proliferation and survival of infected cells despite host immune defenses [1,2].

KS is recognized as an AIDS-defining illness, signifying significant immune suppression [3]. The advent of combination antiretroviral therapy (cART) has markedly decreased the incidence of KS among people with HIV (PWH) [3,4]. However, KS rates remain disproportionately elevated among certain subpopulations, especially men who have sex with men (MSM), likely due to a higher prevalence of KSHV infection in this group [3]. Among cART-treated MSM, CD4 count remains the most significant predictor of KS risk, with incidence rates dramatically higher in those with CD4 counts below 200 cells/mm³ compared to those with higher counts [5].

Clinically, cutaneous KS typically presents as purplish, red-blue, or brown-black macules, papules, or nodules, which may bleed or ulcerate [1]. A definitive diagnosis requires a skin biopsy demonstrating characteristic vascular proliferative features [2]. If untreated, KS can progress from localized skin lesions to extensive visceral involvement, resulting in substantial morbidity and complicating HIV management [6].

First-line treatment for early-stage AIDS-related KS involves initiating or optimizing cART, which alone often leads to tumor regression. In cases of advanced or disseminated KS, systemic chemotherapy, most commonly with pegylated liposomal doxorubicin or paclitaxel, may be indicated, although responses are typically palliative rather than curative [2,6].

Preventing KS hinges on preventing KSHV transmission and ensuring effective HIV control through sustained cART adherence. Despite improved antiretroviral therapy (ART) access, adherence and viral suppression remain suboptimal among racial and ethnic minority populations, particularly Black MSM, due to a constellation of structural, psychosocial, and individual-level barriers [7].

Here, we present the case of a 36-year-old African American MSM with HIV/AIDS residing in an underserved community, who developed disseminated KS following six months of non-adherence to cART.

## Case presentation

A 36-year-old African American MSM, with a history of HIV infection and late latent syphilis, was admitted to our safety-net hospital in December 2024 through the emergency department with complaints of a chronic cough and multiple skin lesions. He reported experiencing a chronic, productive cough with brown phlegm for several months prior to this admission, occasionally accompanied by blood-streaked sputum following episodes of intense coughing. He endorsed night sweats but denied fever, chills, significant weight loss, diarrhea, abdominal pain, or dysuria. Approximately one month prior, during a clinic visit at another institution, he underwent a chest X-ray and was diagnosed with pneumonia, for which azithromycin was prescribed. Despite treatment, his symptoms persisted. The patient reported being diagnosed with HIV-1 infection in 2017, although no documentation or treatment records are available to confirm this diagnosis. Reportedly, he was also diagnosed with syphilis and received three intramuscular (IM) injections of penicillin. He reported a history of three prior hospitalizations since 2017 for recurrent rectal abscesses, each requiring surgical drainage and antibiotic therapy. He was first seen at our Infectious Disease (ID) clinic in October 2023, when his CD4 count was 44 cells/mm³ (reference range: 500-1500 cells/mm³). He was initiated on bictegravir/emtricitabine/tenofovir alafenamide (Biktarvy) with prophylactic sulfamethoxazole/trimethoprim and received three weekly IM penicillin injections for late latent syphilis. However, the patient was subsequently lost to follow-up for approximately one year, during which both the hospital and pharmacy were unable to contact him. He reported non-adherence to both Biktarvy and sulfamethoxazole/trimethoprim for the past six months. He noted mild pruritus after taking Biktarvy, which resolved with continued use, and stated he could tolerate the medication but had difficulty with “mental commitment” to daily therapy.

He recalled noticing a small dark spot on the left upper chest wall approximately one year prior to this hospitalization, followed by the appearance of additional lesions in various areas that had grown rapidly over the past few months.

**Figure 1:**
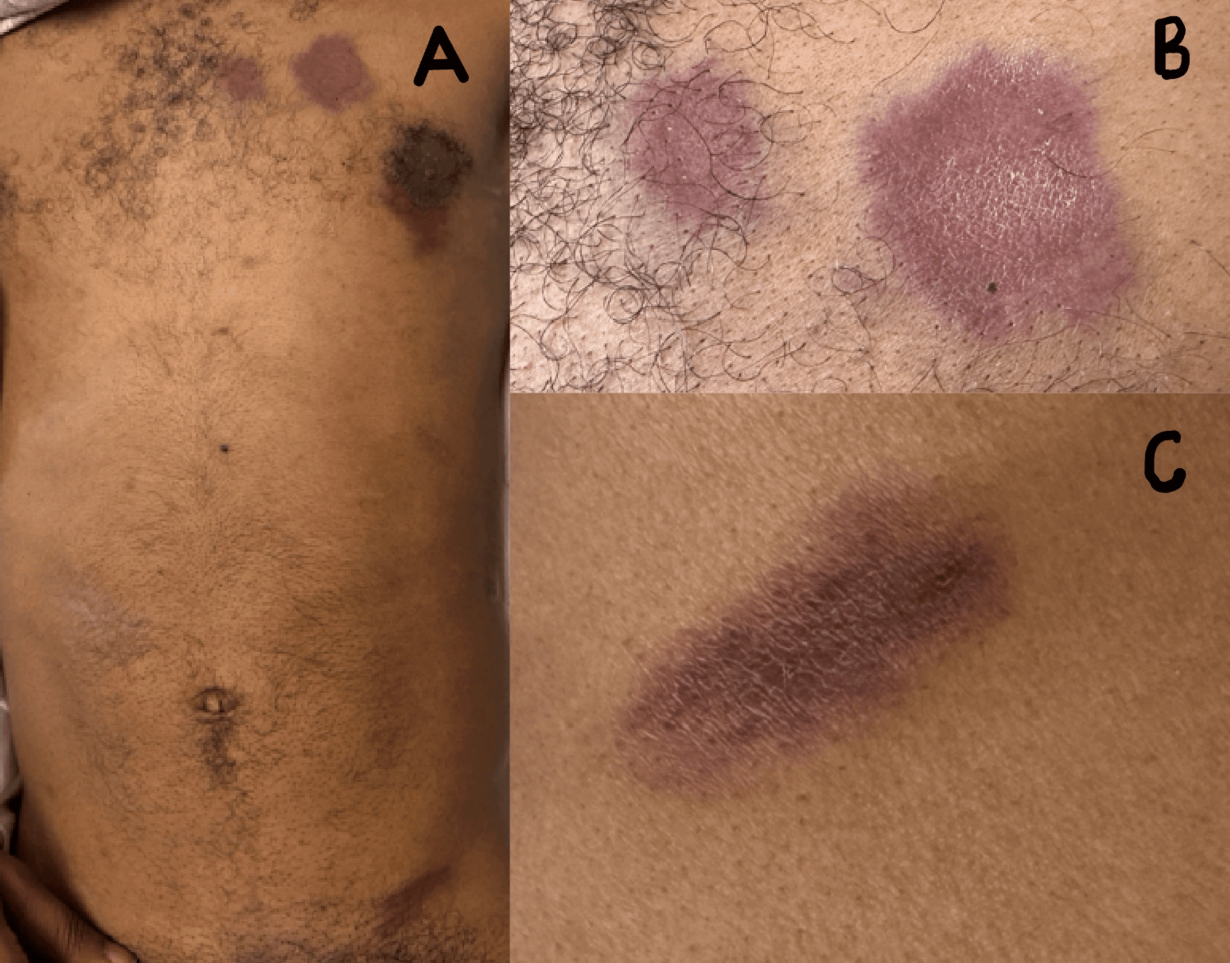
(A) Reddish-purple papules and macules in the left anterior chest and left groin; (B) close-up image of the left anterior chest skin lesions; (C) close-up view of the left flank skin lesion

On admission, vital signs were as follows: blood pressure 142/95 mmHg, heart rate 81 bpm, respiratory rate 18 breaths per minute, and oxygen saturation 99% on room air. Physical examination revealed a chronically ill-appearing, wasted individual with multiple painless, non-blanching, reddish-purple papules and macules on the left anterior chest wall, left groin, back, and flank (Figures 1A-1C).

The chest wall lesions measured 1.5 × 2 cm, 3 × 3 cm, and 3 × 4 cm, while the lesion in the left groin measured 2 × 4 cm. On the left lateral flank, two lesions measured 1.5 × 3.5 cm and 1 × 1 cm, respectively. On the lower back, there were two lesions measuring 3 × 4 cm and 1 × 2 cm. A hematology and oncology consultation was obtained to evaluate suspected KS.

**Figure 2:**
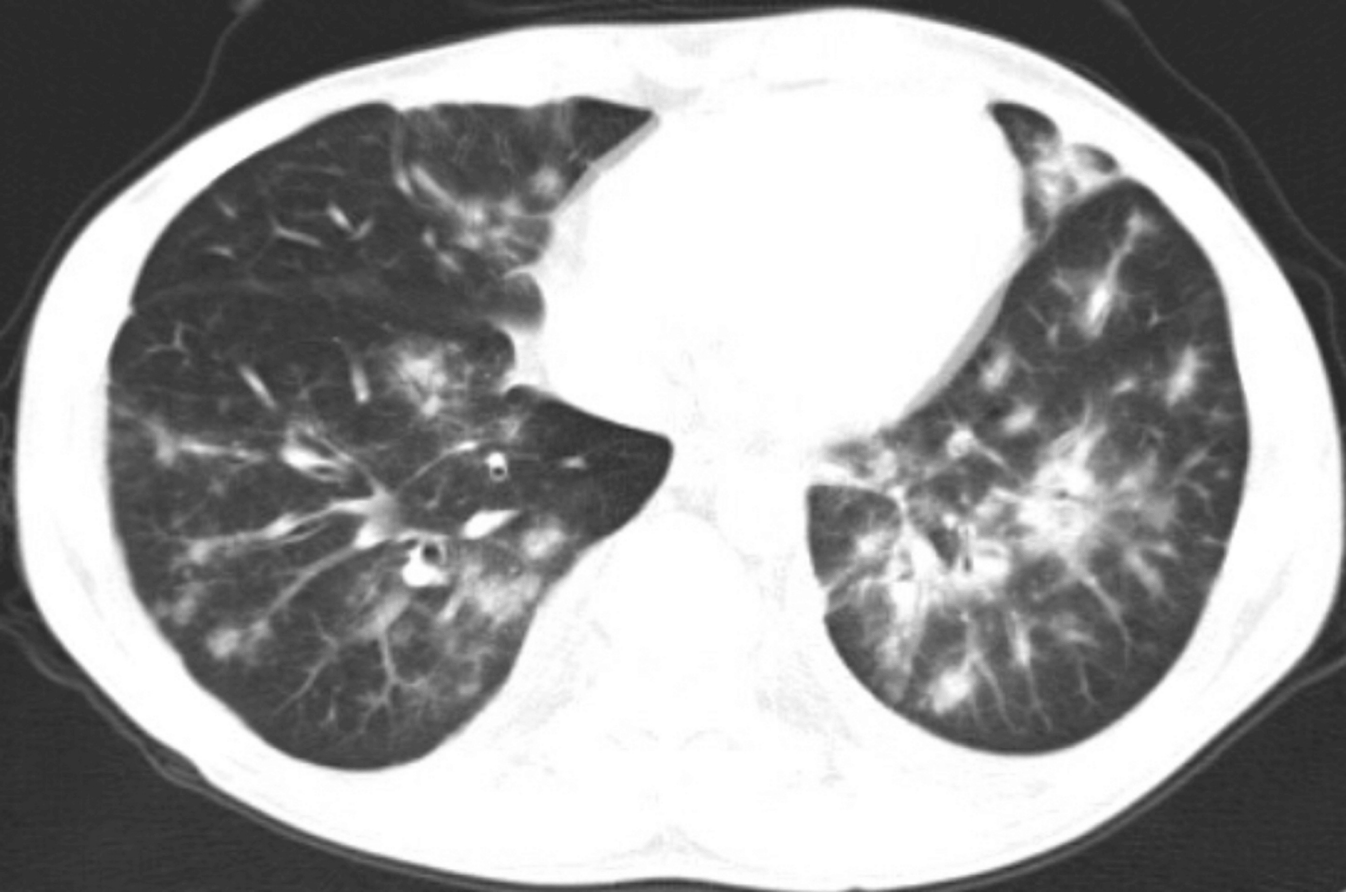
CT scan of the chest showing diffuse bilateral reticulo-nodular opacities and infiltrates, a characteristic of pulmonary Kaposi sarcoma

**Figure 3:**
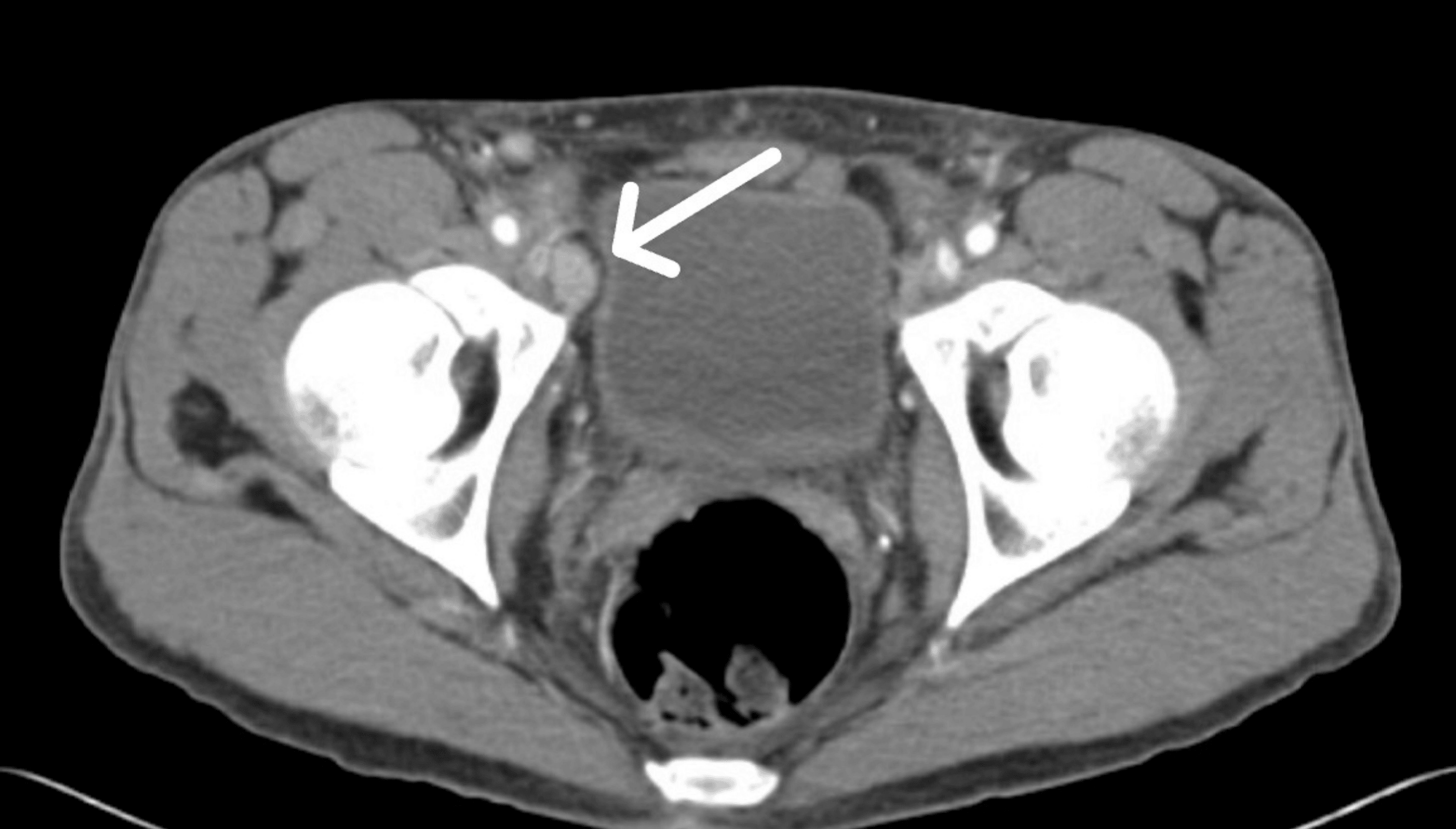
CT scan of the pelvis demonstrating right external iliac lymph node enlargement (arrow)

A contrast-enhanced computed tomography (CT) scan of the chest, abdomen, and pelvis revealed diffuse bilateral reticulonodular infiltrates, a characteristic of pulmonary KS (Figure 2), most confluent in the left upper and lower lobes, mild bilateral pleural effusions, and enlarged right external iliac lymph node (Figure 3) and bilateral inguinal lymphadenopathy.

**Figure 4:**
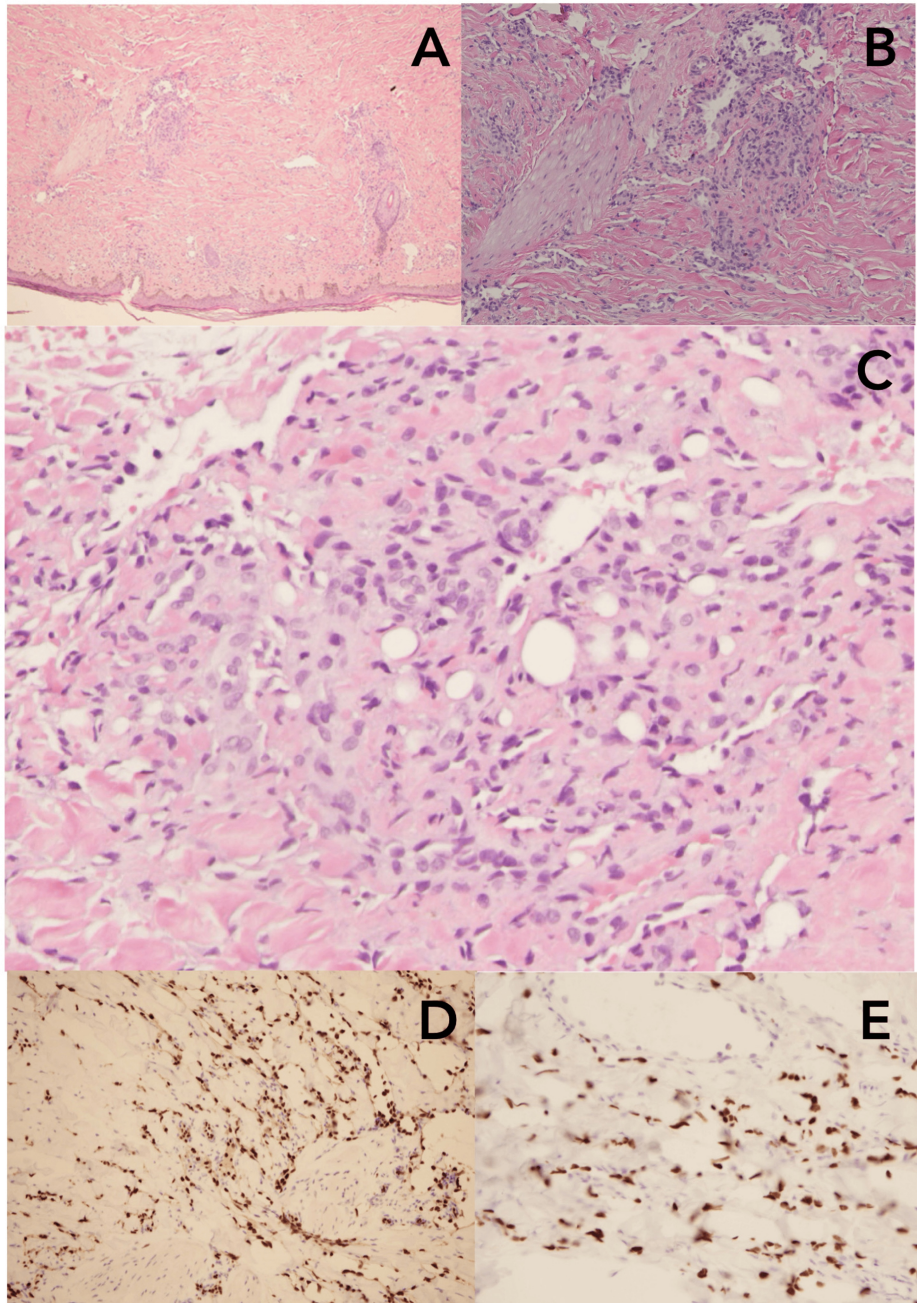
(A) Hematoxylin and Eosin (H&E) stain of skin lesion showing Kaposi sarcoma (KS) (4×); (B) H&E stain of skin lesion showing KS (10×); (C) H&E stain of skin lesion showing KS (20×); (D) immunohistochemistry (IHC) stain of skin lesion positive for ETS-related gene (ERG); (E) IHC stain of the skin lesion positive for human herpesvirus (HHV)-8

Initial laboratory studies were notable for a white blood cell count of 3.5 × 10³/µL with mild eosinophilia (12.4%), red blood cell count of 4.23 × 10⁶/µL, reactive HIV-1/2 antigen/antibody combination test, and an HIV viral load of 592,000 copies/mL. Syphilis antibody testing was reactive, with an RPR titer of 1:2. Flow cytometry confirmed a CD4 count of 44 cells/µL. Differential diagnosis included tuberculosis, pulmonary *Pneumocystis jirovecii*, and bacterial or fungal infection. However, QuantiFERON-TB Gold and coccidioidomycosis serologies, as well as sputum culture, were negative. Bronchoscopy with bronchoalveolar lavage was not done. An incisional biopsy of a skin lesion confirmed the diagnosis of KS (Figures 4A-4E).

The patient was discharged on Biktarvy 50 mg/200 mg/25 mg daily, sulfamethoxazole/trimethoprim 800 mg/160 mg daily, and promethazine as needed for nausea. He was given an appointment with the hematology and oncology clinic and the ID clinic.

## Discussion

Although the patient has been restarted on ART, the long-term outcome remains uncertain. This case highlights the severe consequences of ART non-adherence in a patient from an underserved community, which culminated in the development of disseminated KS. Despite previous engagement in care and initiation of effective ART, lapses in follow-up and medication adherence led to profound immunosuppression and the emergence of advanced HIV-associated malignancy. This underscores the multifactorial nature of ART noncompliance, often driven by structural, psychosocial, and behavioral barriers that disproportionately affect marginalized populations.

In the United States, fewer than half of individuals diagnosed with HIV remain consistently engaged in care, and only about 25% of those achieve sustained viral suppression [8]. Data from a recent retrospective Medicare cohort revealed that treatment gaps of seven days or more and 30 days or more were present in 55.2% and 26.2% of patients, respectively, with 10.1% permanently discontinuing therapy [9]. These findings reflect broader systemic challenges, including stigma, mental health burden, and lack of continuity in healthcare access, particularly among Black MSM and others facing compounded disparities [6,7].

Traditional adherence strategies emphasizing education and counseling remain essential, but they are often insufficient in isolation. Innovative approaches are urgently needed to address adherence in resource-limited environments. Long-acting injectable ART, such as cabotegravir/rilpivirine, offers a promising alternative to daily oral therapy, particularly for individuals experiencing pill fatigue or stigma-related barriers [10,11]. Similarly, artificial intelligence-driven monitoring tools and telehealth-based services have improved engagement, medication tracking, and patient-provider communication in real-time, even in remote or underserved regions [12]. Just-in-time adaptive interventions represent a novel strategy by delivering behavioral prompts and adherence support precisely when patients are most vulnerable to missing doses, based on contextual and digital behavior data [13,14].

Collectively, these advances represent a shift toward precision-based public health interventions designed to be scalable, personalized, and responsive to the needs of the most vulnerable populations. As demonstrated in this case, failure to address ART nonadherence with innovative and inclusive strategies can result in devastating health consequences for patients already at elevated risk due to social and systemic inequities.

## Conclusions

This case underscores the critical importance of sustained adherence to ART in preventing the progression of HIV and its associated complications, including KS. It also emphasizes the urgent need for healthcare systems to go beyond traditional models and implement precision-driven, multidisciplinary strategies to support adherence, especially within vulnerable and underserved communities. Addressing barriers to care, enhancing patient engagement, and expanding access to long-acting therapies and real-time digital interventions are essential steps toward reducing disparities and improving long-term outcomes for people living with HIV.
